# A Real-Time Infrared Ultra-Spectral Signature Classification Method via Spatial Pyramid Matching

**DOI:** 10.3390/s150715868

**Published:** 2015-07-03

**Authors:** Xiaoguang Mei, Yong Ma, Chang Li, Fan Fan, Jun Huang, Jiayi Ma

**Affiliations:** 1 School of Electronic Information and Communications, Huazhong University of Science and Technology, Wuhan 430074, China; E-Mails: meixiaoguang@hust.edu.cn (X.M.); changli@hust.edu.cn (C.L.); fanfan@hust.edu.cn (F.F.); 2 Electronic Information School, Wuhan University, Wuhan 430072, China; E-Mails: junhwong@whu.edu.cn (J.H.); jiayima@whu.edu.cn (J.M.)

**Keywords:** ultra-spectral signature, classification, spatial pyramid matching

## Abstract

The state-of-the-art ultra-spectral sensor technology brings new hope for high precision applications due to its high spectral resolution. However, it also comes with new challenges, such as the high data dimension and noise problems. In this paper, we propose a real-time method for infrared ultra-spectral signature classification via spatial pyramid matching (SPM), which includes two aspects. First, we introduce an infrared ultra-spectral signature similarity measure method via SPM, which is the foundation of the matching-based classification method. Second, we propose the classification method with reference spectral libraries, which utilizes the SPM-based similarity for the real-time infrared ultra-spectral signature classification with robustness performance. Specifically, instead of matching with each spectrum in the spectral library, our method is based on feature matching, which includes a feature library-generating phase. We calculate the SPM-based similarity between the feature of the spectrum and that of each spectrum of the reference feature library, then take the class index of the corresponding spectrum having the maximum similarity as the final result. Experimental comparisons on two publicly-available datasets demonstrate that the proposed method effectively improves the real-time classification performance and robustness to noise.

## 1. Introduction

According to Hunt's research [1], for earth materials, the typical half depth of absorption peaks ranges from 20 nm to 40 nm. The hyperspectral sensors can offer high spectral resolution, which is very important for many applications, such as land use analysis, environmental studies, military surveillance, food quality control, and so on [2,3]. The spectral resolution of them has achieved less than 10 nm, which enables precise classification among different materials. The classification technology is currently the predominate method for analyzing hyperspectral images and has received much attention. However, in some applications, such as gas spectral signature analysis [4,5], it requires even higher spectral resolution, e.g., 1 cm^−1^ (the unit “cm^−1^” represents wavenumber resolution, and the corresponding wavelength resolution differs for each band; for example, at the typical mid-infrared waveband 3 μm, the corresponding wavelength resolution is 0.9 nm). Fortunately, the state-of-the-art ultra-spectral sensor technologies [6], for example the tropospheric emission spectrometer (TES) [7] and the infrared atmospheric sounding interferometer (IASI) [8], have achieved a spectral resolution less than 0.5 cm^−1^, which brings new hope for those high precision applications. Nevertheless, the breakthrough of spectral resolution leads to some new problems, which makes spectral signature analysis become more challenging, especially in real-time applications, such as greenhouse gases surveillance [9,10], target detection [11,12], *etc.*

Higher spectral resolution usually leads to a higher dimension of data, which is very challenging to those real-time required applications mentioned beforehand. For example, typical airborne visible/infrared imaging spectrometer (AVIRIS) hyperspectral data have 224 bands, while TES ultra-spectral data have 9000 specific bands [13]. The conventional methods for spectral signature classification are based on the Euclidean distance (ED) or Hamming distance (HD). In practice, a spectral library is needed for classification. By calculating the ED/HD between the observed spectrum and each spectrum of the library, the classification result can be obtained by searching the minimum of the ED/HDs. Although the conventional methods are easy to implement, the time consumption is huge for hyper-/ultra-spectral signature classification.

For hyper-/ultra-spectral sensors, the signal-to-noise ratio (SNR) is in direct proportion to the square root of the scan time *t*, the spectral resolution Δ*ν* and the radiation flux *E*, *i.e.*, SNR ∝ $\sqrt{t}\Delta vE$ [14]. As the spectral resolution improves, the SNR is decreased with a constant radiation flux and scan time. In low SNR situations, the ED between detected spectra and real spectra increases, which probably degrades the performance of ED/HD matching-based classification algorithms. Although a pre-processing is usually used to deal with spectral noise [15,16], the pre-processing can not always perform well in different situations. Therefore, how to address the problem that the classification accuracy decreases due to the improvement of spectral resolution is another critical issue in ultra-spectral signature classification.

The traditional hyperspectral signature classification algorithms based on distance matching and waveform prediction have low complexity and good classification accuracy. However, as the spectral resolution improves, these methods have severe drawbacks, especially the huge time consumption. This is because the traditional methods perform in a band-to-band fashion without considering the redundancy between each band. Since the high number of bands causes dimensionality problems, a dimensionality reduction (DR) of the hyperspectral vectors can highly facilitate the analysis afterwards [8]. The feature extraction and band selection are typical DR methods, which can significantly reduce the time consumption in the classification process. However, the sensor, photon effects and calibration error [17] unavoidably introduce noises into the acquired hyper-/ultra-spectral data and, thus, degrade the accuracy of the classification performance.

There is a continuous demand for reducing time consumption and improving the accuracy of classification algorithms [18,19]. The spatial pyramid matching (SPM) method [20] has been demonstrated as an excellent feature extraction method and is widely used in image feature extraction [21,22] and image classification [23,24]. However, to the best of our knowledge, there is no report that the SPM-based method has been used for ultra-spectral signature classification. In this paper, to address the above-mentioned issues, we propose an ultra-spectral classification method based on SPM, which includes the following two aspects. First, we introduce an infrared ultra-spectral signature similarity measure via SPM, which is the basis of the matching-based classification method. Second, we propose the classification method with reference spectral libraries, which utilize the SPM-based similarity for real-time and robust infrared ultra-spectral signature classification. Specifically, we divide the spectrum along the wavenumber axis into several sub-blocks. The histogram of each sub-block is calculated by the SPM, which is taken as the elemental feature of a spectrum. To generate the histogram of each sub-block, we quantize the spectral radiation values and choose a proper quantile interval to suppress the negative effect of noise. As the feature of a spectrum, the histograms usually have much lower dimension than that of the original spectrum, which achieve the goal of dimensionality reduction for the ultra-spectral data. When the histograms of a spectrum are obtained, we use the introduced similarity measure method for classification purpose. For real-time applications, instead of matching with each spectrum of the spectral library, our method is based on feature matching, which includes a feature library-generating phase. We calculate the SPM-based similarity between the feature of the spectrum to match and that of each spectrum of the reference feature library, then take the class index of the corresponding spectrum having the maximum similarity as the final result.

Our main contribution in this paper is that we propose a real-time ultra-spectral signature classification method, which mainly includes the similarity measure and the real-time classification method via SPM with reference spectral libraries. Compared with the other four methods, our method can significantly improve the real-time and classification performance in ultra-spectral signature classification applications, especially under a low SNR environment.

## 2. Related Work

Over the past years, two general approaches have been investigated for hyperspectral signature characterization [25]. The first is a coding-based approach, which encodes spectral signatures as code words. Then, spectral analysis is conducted by using the HD as a spectral similarity measure. A typical example is binary coding (BC) [26]. It compares the radiation value of each waveband with a threshold and then transforms the comparison results into binary numbers. The algorithm has very low complexity. However, due to the significant error in quantization process, it may lose some important spectral information. Moreover, it cannot classify spectra within the same class, due to the homogeneity of spectral signatures in the same class [27]. To address these problems, other algorithms were proposed, for example the spectral analysis manager (SPAM) [28], the spectral feature-based binary coding (SFBC) [29], the spectral derivative feature coding (SDFC) [25], *etc*. All of these algorithms use extra bits to encode spectral features, such as spectral derivatives and neighboring waveband differences, which can carry more spectral information. The second type of approach is a signature estimation-based approach, which estimates spectral profiles for signatures, and spectral analysis is then carried out by using the commonly-used least squares error as a criterion for optimality, such as methods based on wavelets and the Kalman filter [25]. The above-mentioned two kinds of methods have shown their good performance in multispectral or even hyperspectral applications. However, for ultra-spectral technology, with its higher spectral resolution, the traditional methods cannot meet the needs in real-time and robust detection well, such as greenhouse and target detection applications.

To achieve the goal of dimensionality reduction, other feasible methods have been investigated. A typical DR method is the band selection. For instance, the mutual information (MI)-based [30] method calculates the MI between observed spectra and the reference spectral library, then selects the bands with the relatively higher MI. The adaptive band selection (ABS) [31] selects the bands with the largest possible information and the least correlation among them. Although those methods can reduce the time consumption in the classification process, they need a large number of observed spectra for each matching process, which cannot meet the needs of real-time classification. Recently, Fang *et al.* proposed the crosscut feature extraction matching method (CF) [13], which can rapidly fulfill the matching process and be implemented in real-time with excellent classification accuracy. However, it uses the crosscut intersections as the feature and adopts the ED for matching, which could make the accuracy decrease due to its sensitivity to noise. Besides, some feature matching methods [32–35] in the computer vision area can also be generalized for spectral matching, but they have a prerequisite that the spectral features should be extracted in advance.

## 3. The Spatial Pyramid Matching Kernel

In this section, we introduce the original formulation of the spatial pyramid matching kernel commonly used in image processing, which is the theoretical foundation of our work. The spatial pyramid matching kernel is based on the histogram intersection kernel, which is also known as the min kernel and has been widely used in image classification [36–40]. The histogram intersection kernel function is shown as follows [37]:

(1) $$I(\text{a},\text{b})=\overset{n}{\underset{i=1}{\text{∑}}}\min(a_{i},b_{i})$$

where ***a*** and ***b*** denote two vectors with the dimension of *n* and *a_i_* and *b_i_* denote the *i*-th entries of ***a*** and ***b***, respectively. In image processing, vectors ***a*** and ***b*** can be taken as the histograms of two images. Thus, when the images are divided into different indexed sub-blocks, *a_i_* and *b_i_* are referred to as the *i*-th histograms of the *i*-th sub-blocks.

Graumann and Darrell [41] proposed the pyramid matching method to find an approximate correspondence between two vectors. However, the pyramid scheme works with an orderless image presentation and discards all spatial information. To address this issue, Lazebnik *et al.* [20] proposed a spatial pyramid scheme to utilize the spatial information of data. In this paper, we regard the inter-bands relationship as the corresponding spatial information of a spectrum. The spatial pyramid matching works by placing a sequence of increasingly coarser cells over the feature space and taking a weighted sum of histogram intersection kernels that occur at each level of spatial resolution. Those cells are generated by repeatedly subdividing along the wavenumber axis into different cells at increasingly fine resolutions. More specifically, *x* and *y* denote two spectra of the same dimension *N*. 0,1,…, *L* denote the resolution levels from coarse to fine, where *L* <= log_2_ *N*. At level *l*, the two spectra are divided along the wavenumber axis into *2^l^* cells, which are of the same length. Then, the histograms of each cell for the two spectra are calculated, where $H_{x}^{l}(i)$ and $H_{y}^{l}(i)$ denote the histograms of the *i*-th cells at the resolution level *l*. Thus, the histogram intersection kernels can be calculated by [20]:

(2) $$I(H_{x}^{l},H_{y}^{l})=\overset{D}{\underset{i=1}{\text{∑}}}\min(H_{x}^{l}(x_{i}),H_{y}^{l}(y_{i}))$$

where *D* (*D* = 2*^L^*) denotes the number of cells. Since the coarser resolution levels include more dissimilar features, it is reasonable to penalize matches found at the coarser levels. Then, we get the following definition of a spatial pyramid matching kernel [20]:

(3) $$\begin{array}{ll} \kappa^{L}(\text{x},\text{y}) & =I(H_{x}^{L},H_{y}^{L})+\overset{L-1}{\underset{l=0}{\text{∑}}}\frac{1}{2^{L-l}}(I(H_{x}^{l},H_{y}^{l})-I(H_{x}^{L},H_{y}^{L}))\\ & =\frac{1}{2^{L}}I^{0}+\overset{L}{\underset{l=1}{\text{∑}}}\frac{1}{2^{L-l+1}}I^{l} \end{array}$$

where *I*^0^ and *I^l^* represent the histogram intersection kernels at the resolution levels 0 and *l*, respectively, which can be obtained according to Equation (2).

## 4. The Infrared Ultra-Spectral Signatures Similarity Measure Method via SPM

In this paper, we use a matching-based method to classify the spectra, which needs the criterion of a similarity measure. Based on the spatial pyramid matching kernel mentioned above, we introduce the following method for measuring the similarity of the infrared ultra-spectral signatures. Specifically, first, we quantize the spectral value of ***x*** and ***y*** into *M* discrete levels. Second, we calculate the histogram intersection kernel at each discrete level and the spatial pyramid matching kernel according to Equations (2) and (3), respectively. In detail, for each resolution level *l*, we divide the total *N* bands into 2*^l^* sub-blocks and calculate the corresponding histograms. Then, the histogram intersection kernel *I_l_* can be obtained according to Equation (2). Specially, *I*_0_ represents histogram intersection at the coarsest spatial resolution level, *i.e.*, the histogram intersection across the entire bandwidth. When we get *I_l_* and *I*_0_, the spatial pyramid kernel can be obtained according to Equation (3). Finally, we use *K^L^* as the classifier, which can be obtained by summing up each entry of the spatial pyramid kernel. The classifier *K^L^* can be expressed as follows:

(4) $$K^{L}(\text{x},\text{y})=\overset{M}{\underset{m=1}{\text{∑}}}\kappa_{m}^{L}(x,y)$$

where $\kappa_{m}^{L}$ denotes the *m*-th entry of the spatial pyramid kernel vector *κ^L^*. The higher the *K^L^* is, the more similar the two spectra ***x*** and ***y*** are. Note that we do not combine the kernel Equation (4) with SVM like [20] does. This is because, once the supervised training joins in, the real-time performance could be sacrificed. Additionally, supervised classifiers require a large quantity of labeled data due to the high dimensional spectral data vector. However, labeled instances are often difficult, costly or time consuming to obtain [42].

The detailed procedures of the method can be referred to in Algorithm 1.


**Algorithm 1:** The similarity measure via spatial pyramid matching (SPM).
**Input:** measure_simi(*v*_1_, ⋯ *v_N_*, *r*_1_, ⋯ *r_N_*, *L*, *M*) % *v_i_* and *r_i_* denote the two spectra.**Output:** The pyramid matching kernel *K^L^*1Initialize *κ^L^* = **0**_1×_
*_m_*, *I*_0_ ∼ *I_L_* = **0**_1×_*_M_*;2**for**
*l* = 0, ⋯ ,*L*
**do**3  *D_l_* = 2*^l^*; % divide *N* into *D_l_* parts.4  *dim_l_* = *N*/2*^l^*; % the dimension of parts belonged to the *l*-th level.5  **for**
*i* = 1, ⋯ ,*D_l_*
**do**6   % *ha_i_* and *hb_i_* denote the *i*-th sub-blocks of the two spectra.7   *ha_i_* = calculate the histogram from *v*_((_*_i_*_-1)*__dim_l___+1)_ to v_i__*__dim_l__;8   *hb_i_* = calculate the histogram from *r*_((_*_i_*_-1)*__dim_l___+1)_ to *r*_i*dim_l__;9   *I_l_* = *I_l_* + min(*ha_i_*, *hb_i_*);10  **end**11**end**12calculate *κ^L^* according to Equation (3);13calculate *K^L^* according to Equation (4);


For better understanding, we choose two spectra for illustration. These two spectra are named as Spectrum A and Spectrum B (Spec. A and Spec. B), respectively. Both of them have the same dimension of *N*. The spectral quantile level *M* is set to 10 for the histogram calculation. For simplicity, when *L* = 2, the spatial pyramid will have three levels, *i.e.*, *l* = 0, 1, 2. The procedures are shown with the following steps.

**Figure 1:**
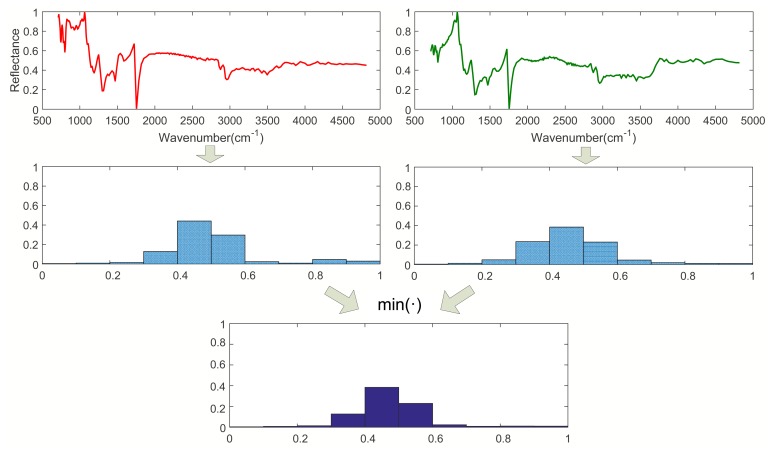
The spatial pyramid at Level 0 (*l* = 0). The first row: Spectrum A (left), Spectrum B (right). The second row: the histogram of Spec. A (left), the histogram of Spec. B (right). The third row: the histogram intersection kernel referred to in Equation (2).

**Figure 2:**
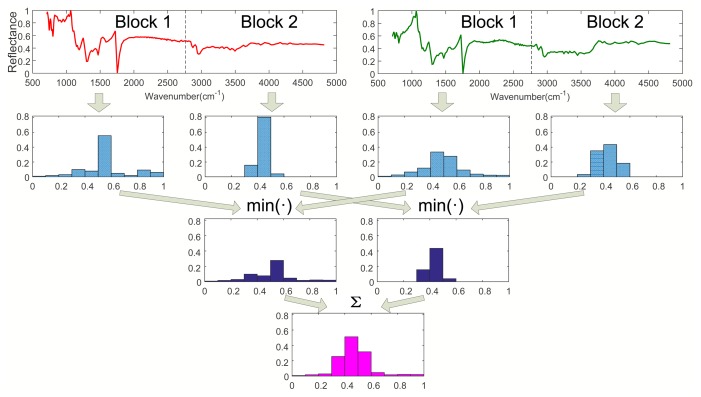
The spatial pyramid at Level 1 (*l* = 1). The first row: the two spectra (Spectrum A (left), Spectrum B (right)) are divided into two sub-blocks, respectively. The second row (from the left to the right): the histogram of Block 1, Spec. A, the histogram of Block 2, Spec. A, the histogram of Block 1, Spec. B, the histogram of Block 2, Spec. B. The third row: the histogram intersection kernels. The fourth row: the summation of the histogram intersection kernels referred to in Equation (2).

**Figure 3:**
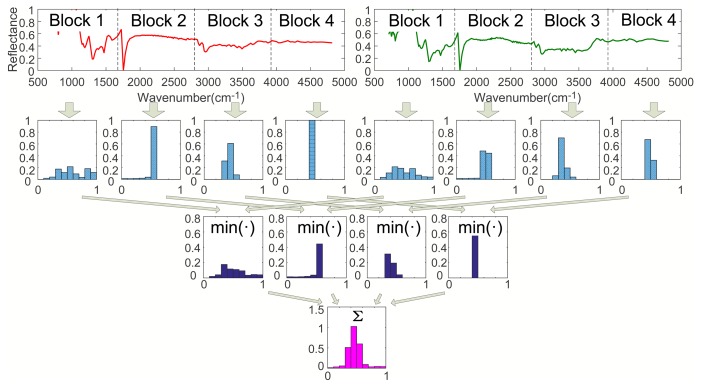
The spatial pyramid at Level 2 (*l* = 2). The first row: the two spectra (Spectrum A (left), Spectrum B (right)) are divided into four sub-blocks, respectively. The second row (from the left to the right): the histogram of Block 1, Spec. A, the histogram of Block 2, Spec. A, the histogram of Block 3, Spec. A, the histogram of Block 4, Spec. A, the histogram of Block 1, Spec. B, the histogram of Block 2, Spec. B, the histogram of Block 3, Spec. B, the histogram of Block 4, Spec. B. The third row: the histogram intersection kernels. The fourth row: the summation of the histogram intersection kernels referred to in Equation (2).

- Step 1. Calculating *I*_0_ (*l* = 0):
  As shown in Figure 1, when *l* = 0, calculate the histograms of Spectra A and B with the entire wavebands. Then, calculate *I*_0_ according to Equation (2), where *I*_0_ is a vector with *M* dimension.
- Step 2. Calculating *I*_1_ (*l* = 1):
  As shown in Figure 2, when *l* = 1, divide Spectra A and B into two sub-blocks with the same length, respectively. Calculate the histograms of all sub-blocks of Spec. A and B. Then, obtain two minimum vectors by comparing the histograms of all parts of Spec. A with that of Spec. B, respectively. Finally, we have *I*_1_ by summing up the two minimum vectors, where *I*_1_ is a vector with *M* dimension.
- Step 3. Calculating *I*_2_ (*l* = 2):
  As shown in Figure 3, when *l* = 2, divide Spectra A and B into four sub-blocks with the same length, respectively. Calculate the histograms of Block 1 to 4 of Spec. A and B. Then, obtain four minimum vectors by comparing the histograms of all sub-blocks of Spec. A with that of Spec. B, respectively. Finally, we have *I*_2_ by summing up the four minimum vectors, where *I*_2_ is a vector with *M* dimension.
- Step 4. Calculating *κ^L^*:
  We get *κ^L^* by the weighted sum of *I*_0_, *I*_1_, *I*_2_ according to Equation (3), where *κ^L^* is a vector with *M* dimension.
- Step 5. calculating *K^L^*:
  Calculate *K^L^* according to Equation (4).

## 5. The Proposed Real-Time Infrared Ultra-Spectral Signature Classification Method via SPM with Reference Spectral Libraries

### 5.1. Methodology

In practice, the matching-based ultra-spectral signature classification methods are commonly based on reference spectral libraries. Usually, the libraries have a large number of spectra with a high dimension. Moreover, the observed spectra to be matched are usually corrupted by the environmental noise. To achieve better performance, we propose the real-time infrared ultra-spectral signature classification method via SPM, which is based on the following considerations.

The CF [13] method just uses the cross lines to extract the spectral features. However, it is sensitive to noise, and the classification performance decreases when the spectra are degraded by noise. To make it robust to the noise, the spatial pyramid kernels we used are based on the histograms. As we analyzed, a proper quantization process can improve the tolerance for the noise. The larger the quantile interval is, the more robust the method for noise is.

However, an improper selection of quantile interval could bring quantile error for the method. Specifically, it is assumed that the quantile error is uniform distributed in the quantile interval $\left[-\frac{q_{\text{interval}}}{2},+\frac{q_{\text{interval}}}{2}\right]$; thus, the variation of quantile error is:

(5) $$\sigma_{QE}^{2}=\int_{-q_{\text{interval}}/2}^{+q_{\text{interval}}/2}e^{2}p(e)de=\int_{-q_{\text{interval}}/2}^{+q_{\text{interval}}/2}e^{2}\frac{1}{q_{\text{interval}}}de=\frac{q_{\text{interval}}^{2}}{12}$$

where *e* denotes the quantile error and $p(e)=\frac{1}{q_{\text{interval}}}$ is the probability density function of the quantile error. To obtain the SNR, we have to calculate the uniformed signal power:

(6) $$s^{2}={\left(\frac{M\cdot q_{\text{interval}}}{2}\right)}^{2}=\frac{M^{2}\cdot q_{\text{interval}}^{2}}{4}$$

where *M* denotes the number of quantile levels. The SNR can be computed as follows:

(7) $$\text{SNR}=\frac{s^{2}}{\sigma_{QE}^{2}}=\frac{M^{2}\cdot q_{\text{interval}}^{2}/4}{q_{\text{interval}}^{2}/12}=3M^{2}$$

According to Equation (7), it is obvious that SNR is in direct proportion to *M*. It is also shown that when quantile levels increase, the negative effect of quantile error can be reduced. However, according to the previous analysis in the beginning of this section, when the quantile levels increase, the robustness to noise would decrease. Thus, to achieve good classification performance, we should strike a balance between the quantile error and the robustness to noise, which can be obtained by selecting the optimal quantile level in the following experimental works.

The traditional matching-based classification method does not consider the dimensionality reduction, which would be time consuming. Besides, if a spectral library is directly used as a reference for matching via Algorithm 1, it also cannot meet the real-time needs, because it contains both the feature extraction and matching phases.

In this paper, we achieve the dimensionality reduction of the infrared ultra-spectral signatures via SPM. The reduced dimension is calculated as follows:

(8) $$\text{Dim}=M\cdot \overset{L}{\underset{l=0}{\text{∑}}}2^{l}$$

where *M* denotes the quantile levels and *L* represents the max spatial resolution levels. For example, a spectrum has the dimension of 4287 if we set the quantile level to 10 and the spatial resolution level to 2. Therefore, the total dimension is significantly reduced to 70 according to Equation (8). Meanwhile, we split the feature extraction and matching of Algorithm 1 into two independent phases, the time consumption of which can be significantly reduced because the matching process is based on the features, and the feature library is calculated in advance according to the original spectral library.

According to the considerations mentioned above, we proposed the real-time infrared ultra-spectral signature classification method via SPM, which includes two independent phases. The first one is for spectral feature library generating, and the second one is for spectra matching. The detailed procedures of the 2 phases are shown in Algorithms 2 and 3, respectively.


**Algorithm 2:** The proposed method to generate a spatial pyramid feature library.
**Input:** buildSPfeatlib(***S***, *L*, *M*) % ***S*** denotes the reference spectral library.**Output:** The spatial pyramid *SP_L_* of each spectrum in ***S***1Initialize *SP_L_* = **0**_(1−2^(L+1)^)/(1−2)__×*M*_, *sp_l_* = **0**_2*^l^*__×_
*_M_*, *l* ∈ {0,… , *L*} ;2**for**
*all*
***v*** ∊ ***S***,***v*** = *v*_1_, ⋯ ,*v_N_*
**do**3  **for**
*l* = 0, ⋯ , *L*
**do**4   *D_l_* = 2*^l^*; % divide *N* into *D_l_* parts.5   *dim_l_* = *N*/2*^l^*; % the dimension of parts belonged to the *l*-th level.6   **for**
*i* = 1, ⋯ , *D_l_*
**do**7    *h_i_*= calculate the histogram from *v*_((_*_i_*_−1)*__dim_l___+1)_ to v_i * dim_l__;8    *sp_l,i_* = *h_i_*; % *sp_l,i_* denotes the *i*-th row of *sp_l_*.9   **end**10   **if**
*l* == 0 **then**11    
$SP_{L,2^{l}\sim (2^{\left(l+1\right)}-1)}=\frac{1}{2^{L}}sp_{l}$; % *SP_L,_*_2_*_l∼_*_(2_^_(*l*+1)_^_−1)_ denotes the 2*^l^*-th to 2^(^*^l^*^+1)^ − 1-th rows of *SP_L_*.12   **end**13   **else**14    
$SP_{L,2^{l}\sim (2^{\left(l+1\right)}-1)}=\frac{1}{2^{\left(L-l+1\right)}}sp_{l}$15   **end**16  **end**17**end**


### 5.2. Time Complexity Analysis

The proposed method is matching based, which mainly needs to iteratively extract the features and measure the similarity. Therefore, we regard those iterations as the major contributors to the complexity, for two reasons: the time complexity for feature extraction and similarity calculation. They are denoted as *T_f_* and *T_s_*, respectively.

- Analysis on *T_f_*

**Algorithm 3:** The proposed classification method with a reference spectral feature library.
**Input:** fastSPM(***v***, ***R***, *L*, *M*) % ***R*** denotes the reference spectral feature library.**Output:**
${\text{index}}_{K_{\max}^{L}}$% denotes the matching result and the spectrum index in the library.1Initialize *SP* = **0**_(1−2_^_(*L*+1)_^_)/(1−2)×_*_M_*; % The spatial pyramid feature of the observed spectrum.2Initialize 
$K_{\text{max}}^{L}=0$, 
${\text{index}}_{K_{\text{max}}^{L}}=1$; %
$K_{\text{max}}^{L}=0$ and 
${\text{index}}_{K_{\text{max}}^{L}}=1$ denotes the max kernel and the corresponded index.3*SP* = buildSPfeatlib(***v***, *L*, *M*);4**for** all RSP ∈ ***R* do**5  *κ^L^* = 1_1×(1−2^(*_L_*_+1)_^)__/(1−2)_ · min(*SP*, *RSP*); % *RSP* denotes the spatial pyramid feature of a spectrum.6  *K^L^* = *κ^L^* · 1*_M_*_×1_;7  **if**
$K^{L}>K_{\text{max}}^{L}$
**then**8   
$K_{\text{max}}^{L}=K^{L}$9   assign the index of *RSP* of the library to 
${\text{index}}_{K_{\text{max}}^{L}}$;10  **end**11**end**


According to Algorithm 2, the feature extraction includes the multi-level histogram calculation and other assignment operations. The latter is trivial for the time complexity, and the histogram calculation is the major contributor to the complexity. For a feature extraction scheme with spatial resolution level *L*, we can obtain the highest spatial resolution level (*l* = *L*) histograms, while the lower levels can be simultaneously generated by summation operations. Therefore, the complexity is mainly concentrated on the calculation of the highest level histograms. Suppose we have the quantile level M; the time complexity of feature extraction is:

(9) $$T_{f}=O(MN)$$

where *N* is the spectrum dimension. If we adopt the binary-search method in the quantization process, *T_f_* then becomes:

(10) $$T_{f}=O(\log_{2}MN)$$

Actually, for dimension reduction purpose, *M* is usually small (*M* << *N*). Thus, log_2_ *M* << *N*, and then, we have:

(11) $$T_{f}=O(N)$$

- Analysis on *T_s_*:

When the feature extraction is completed, the output features can be contained by a vector, which has the dimension of $M{\text{∑}}_{l=0}^{L}2^{l}$. According to Algorithm 2, the complexity of the similarity calculation is:

(12) $$T_{s}=O(M\overset{L}{\underset{l=0}{\text{∑}}}2^{l})$$

where $M{\text{∑}}_{l=0}^{L}2^{l}$ is a constant. For dimension reduction purposes, $M{\text{∑}}_{l=0}^{L}2^{l}$ is far smaller than *N*. Thus, the complexity can be expressed by:

(13) $$T_{s}=O(1)$$

Similarly, we investigated the complexity of the four compared methods, including BC, SFBC, SDFC and CF (see Table 1). The results show that CF and the proposed method are superior to the others, especially in the complexity of similarity calculation. Note that we also denote the complexity of the encoding phases of BC, SFBC and SDFC as *T_f_*.

## 6. Experimental Setup

### 6.1. Datasets and Settings

**Figure 4:**
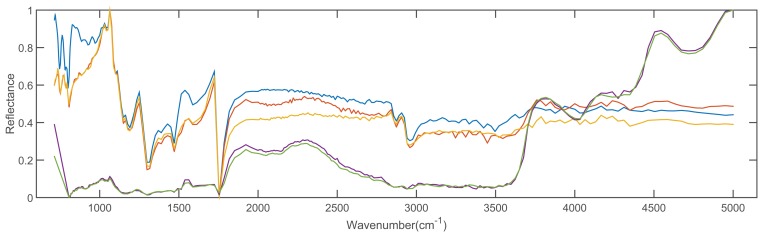
Five spectra of solid man-made materials in the ASTER dataset.

**Figure 5:**
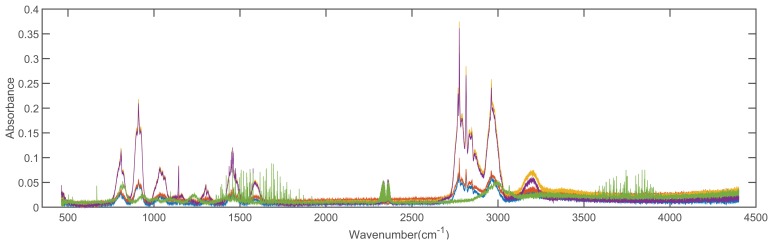
Five spectra of compound 1,1-dimethyl hydrazine with different concentrations in the EPA dataset.

In order to validate the feasibility of the proposed method, we conducted the experiments with 2 spectral libraries (the Advanced Spaceborne Thermal Emission Reflection Radiometer (ASTER) [43] and the Environmental Protection Agency (EAP) [44] spectral libraries), which are taken as the reference spectral libraries. The dimensions of spectra from ASTER and EPA spectral library are 42,861 and 32,000, respectively. There are 1432 types of materials in the ASTER dataset and 384 types of materials in the EPA dataset, respectively. Additionally, we evaluate our method on all of the spectral signatures contained in the datasets. Figure 4 shows the 5 spectra of solid man-made materials in the ASTER dataset, and Figure 5 shows the 5 spectra of compound 1,1-dimethyl hydrazine with different concentrations in the EPA dataset.

The four compared methods are as follows: BC [26], SFBC [29], SDFC [25] and CF [13]. According to the experiments in [13], *M* = 20, *s* = 20 are validated as the optimal settings. Thus, we choose these settings for the following experiments for CF.

An experiment is conducted for the proposed method with different parameter settings to choose the optimal one in advance. In detail, we iteratively pick each spectrum from the two libraries to classify 20 times, respectively. Then, we calculate the average accuracy *η_av_*. To simulate the actual situation in applications, at first, we add noise to the picked spectrum, then we remove the noise according to the algorithm in [45]. The result is shown in Table 2. Therefore, the parameter setting we chosen is *M* = 30, *L* = 3 for the following experiments.

### 6.2. Experimental Method

For each compared method, we iteratively pick each spectrum from the 2 libraries to classify 20 times, respectively. Then, we calculate the average accuracy *η_av_* and average time consumption *t_av_*.

To validate the robustness of all of the methods, before classification, we add different level of noise to the spectra to classify. Specifically, the spectra picked from the 2 spectral libraries are added to the noise of SNR = 45, 50, 55 dB. When noise is added to the spectra, a normalization process is needed. We use the normalization method as follows:

(14) $${\text{v}}_{\text{normalized}}=\frac{\text{v}-\min(\text{v})}{\max(\text{v})-\min(\text{v})}$$

where ***v*** denotes the original spectral vector. max (***v***) and min (***v***) denote the maximal and minimal entry of ***v***, respectively. Besides, to guarantee that the average accuracy results are robust in the sense of statistics, we analyze the standard deviation:

(15) $$\sigma \sqrt{\frac{1}{20}\overset{20}{\underset{i=1}{\text{∑}}}{\left(x_{i}-\mu \right)}^{2}}$$

We use *σ*^ASTER^ and *σ*^EPA^ to denote the standard deviations for ASTER and EPA experiments, respectively. Note that we do not make a similar analysis on time performance experiments, because the time consumption of each algorithm is nearly constant and the deviation is trivial.

## 7. Results and Discussion

### 7.1. Classification Accuracy

Tables 3, 4–5 illustrate the results of the experiments for the accuracy comparisons. The BC method shows good accuracy performance under different noise levels. Here, the BC method we used is the traditional binary coding algorithm with just one threshold, which is set to the mean of the entire spectral radiation value. The BC method also shows good robustness to noise. The accuracy gaps between different noise levels are not sharp. As we know, the main reason to explain why the traditional method has good performance in the experiments is that the noise we added is additive white Gaussian noise, while the threshold of BC uses the mean value to improve SNR, so as to reduce the negative effect of noise. Moreover, the accuracy performance of BC has obvious differences between ASTER and EPA spectral libraries. The results with the ASTER spectral library are better than those of the EPA spectral library. This is because the profiles of ASTER spectra are relatively smoother than those of the EPA's spectra. If the profiles of the spectra have too many steep and large slopes under a low SNR situation, the noise could affect the coding process, which may cause the problem that a correct binary bit could be turned into a false one.

The SFBC method generally has better accuracy performance than the BC. This is because the SFBC includes the BC coding binary bits while using extra binary bits to describe the inter-band information. The extra bits include the slope bits and the mean derivation bits, which describe the slopes between bands and the variations of amplitude about mean derivations, respectively.

The SDFC method has the poorest accuracy performance in the experiments. This is because the SDFC includes the BC method while using extra binary bits to describe the derivative information. The noise we added is additive white Gaussian noise, which could make the derivate bits jitter and disable the extraction of the band-wise information.

The CF shows good results, but without good robustness. It uses a number of equally-spaced horizontal lines to intersect with the spectral curve and takes the total of intersections for each line.

Finally, the total intersections over each line compose a vector, which is taken as the feature of the spectrum. The spectral discrimination between two spectra can be executed by measuring the ED between the feature vectors of the two spectra. Due to the dimensionality reduction via the feature extraction process, this method has low complexity, which can be used in high real-time applications, and it also has decent classification accuracy according to the experiments. However, it has some critical flaws. First, the distance measured by the ED is sensitive to noise, and the threshold needs a sophisticated design. Second, if the spectrum is smooth and relatively monotonous, there may be few intersections, which could a decrease in the robustness of the method.

The experiments for accuracy performance validation demonstrate that the proposed method has the best classification accuracy and robustness in different noise levels for the two datasets. This is because we select the proper quantile levels to improve the robustness to noise, which is discussed in Section 5. Furthermore, the standard deviations at different SNRs are superior to those of other methods, which shows that our method has the highest robustness in the sense of statistics.

### 7.2. Real-Time Performance

Table 6 illustrates the results of the experiments for time consumption comparisons. BC, SFBC and SDFC are point-wise coding scheme-based methods. Thus, every band of the spectrum should be encoded as one or more binary bits (if the dimension of a spectrum is *N*, the encoded binary bits are *N*, 3*N* − 4, 3*N* − 4 via BC, SFBC and SDFC, respectively). Therefore, the data dimensions of spectra are not reduced through these methods, which are time consuming. The CF has good real-time performance, because the data dimension is significantly reduced by the feature extraction. Then, the matching process is conducted based on the feature discrimination, which has low dimension. As the highlight of the method, the time consumption is about 100-times lower than that of BC, SFBC and SDFC. Comparing with CF, the proposed method reduces 0.6% and 35.9% of the time for the EPA and ASTER datasets, respectively.

Note that, according to the analysis in Section 5.2, all methods have the same time complexity in feature extraction phases. Actually, in the experiments, the time consumption of the feature extraction phase of the proposed method *t_s_* is slightly higher than that of BC and CF. This is because our method needs multi-scale processes in both spatial and radiation fields, which takes more time for summation and search operations. In the similarity calculation phase, the proposed method shows its advantages, because the complexity does not increase with *N*. Although CF has the same complexity as the proposed method, the actual time consumption is higher than that of the proposed method. This is because CF needs to calculate the ED, which suffers a lot of multiplication operations, while the proposed method just needs summation operations.

## 8. Conclusions

In this paper, we analyzed the high dimension and noise problems caused by the increase of the spectral resolution in ultra-spectral technology, which probably decreases the classification performance. To address these issues, we proposed a real-time method for infrared ultra-spectral signature classification based on SPM. Experimental comparisons on two publicly-available datasets demonstrate that the proposed method can effectively improve the real-time classification performance (the time consumption is about 100-times lower than that of traditional methods) and robustness to noise.

## Figures and Tables

**Table 1:** Time complexity comparisons. SFBC, spectral feature-based binary coding; SDFC, spectral derivative feature coding; CF, crosscut feature.

**Algorithm**	***T****_f_*	***T****_s_*
BC	*O*(*n*)	*O*(*n*)
SFBC	*O*(*n*)	*O*(*n*)
SDFC	*O*(*n*)	*O*(*n*)
CF	*O*(*n*)	*O*(1)
The proposed method	*O*(*n*)	*O*(1)

**Table 2:** Performance comparisons of the proposed method with different parameter settings.

**Parameter Setting**	$\eta_{av}^{\text{EPA}}(\%)$	$\eta_{av}^{\text{ASTER}}(\%)$
*M* = 10, *L* = 1	91.67	99.23
*M* = 10, *L* = 2	93.75	99.44
*M* = 10, *L* = 3	93.23	99.58
*M* = 20, *L* = 1	91.15	99.37
*M* = 20, *L* = 2	92.19	99.86
*M* = 20, *L* = 3	92.71	99.72
*M* = 30, *L* = 1	92.71	99.51
*M* = 30, *L* = 2	93.49	99.65
***M* = 30, *L* = 3**	**94.01**	**99.93**

**Table 3:** Accuracy comparisons of different algorithms with the 2 datasets and SNR = 45 dB.

**Algorithm**	$\eta_{av}^{\text{EPA}}(\%)$	*σ***^EPA^**(%)	$\eta_{av}^{\text{ASTER}}(\%)$	*σ***^ASTER^**(%)
BC	88.37	0.20	99.16	0.03
SFBC	89.38	0.20	99.58	0.02
SDFC	78.13	0.25	41.90	0.05
CF	79.17	0.10	99.51	0.01
The proposed method	**89.58**	0.10	**99.60**	0.01

**Table 4:** Accuracy comparisons of different algorithms with the 2 datasets and SNR = 50 dB.

**Algorithm**	$\eta_{av}^{\text{EPA}}(\%)$	*σ*^EPA^(%)	$\eta_{av}^{\text{ASTER}}(\%)$	*σ***^ASTER^** (%)
BC	92.45	0.23	98.81	0.03
SFBC	89.84	0.19	99.37	0.03
SDFC	73.44	0.21	43.36	0.02
CF	91.66	0.16	99.59	0.01
The proposed method	**94.01**	0.05	**99.93**	0.01

**Table 5:** Accuracy comparisons of different algorithms with the 2 datasets and SNR = 55 dB.

**Algorithm**	$\eta_{av}^{\text{EPA}}(\%)$	*σ***^EPA^**(%)	$\eta_{av}^{\text{ASTER}}(\%)$	*σ***^ASTER^** (%)
BC	97.40	0.18	99.30	0.02
SFBC	95.83	0.16	99.86	0.02
SDFC	86.46	0.19	45.88	0.03
CF	96.88	0.08	99.72	0.01
The proposed method	**97.40**	0.05	**99.93**	0.00

**Table 6:** Time consumption comparisons of different algorithms with the 2 datasets.

**Algorithm**	$t_{av}^{\text{EAP}}(\text{ms})$	$t_{av}^{\text{ASTER}}(\text{ms})$
BC	2311.85	9422.01
SFBC	2351.65	10,067.12
SDFC	2440.47	9976.25
CF	21.98	58.25
The proposed method	**21.85**	**37.33**
